# Exploration of the *Drosophila buzzatii* transposable element content suggests underestimation of repeats in Drosophila genomes

**DOI:** 10.1186/s12864-016-2648-8

**Published:** 2016-05-10

**Authors:** Nuria Rius, Yolanda Guillén, Alejandra Delprat, Aurélie Kapusta, Cédric Feschotte, Alfredo Ruiz

**Affiliations:** Department de Genética i Microbiologia, Universitat Autònoma de Barcelona, Bellaterra (Barcelona), Spain; Department of Human Genetics, University of Utah School of Medicine, Salt Lake City, UT USA

**Keywords:** Drosophila, Buzzatii, Transposable elements, Genome

## Abstract

**Background:**

Many new Drosophila genomes have been sequenced in recent years using new-generation sequencing platforms and assembly methods. Transposable elements (TEs), being repetitive sequences, are often misassembled, especially in the genomes sequenced with short reads. Consequently, the mobile fraction of many of the new genomes has not been analyzed in detail or compared with that of other genomes sequenced with different methods, which could shed light into the understanding of genome and TE evolution. Here we compare the TE content of three genomes: *D. buzzatii* st-1, j-19, and *D. mojavensis*.

**Results:**

We have sequenced a new *D. buzzatii* genome (j-19) that complements the *D. buzzatii* reference genome (st-1) already published, and compared their TE contents with that of *D. mojavensis*. We found an underestimation of TE sequences in *Drosophila* genus NGS-genomes when compared to Sanger-genomes. To be able to compare genomes sequenced with different technologies, we developed a coverage-based method and applied it to the *D. buzzatii* st-1 and j-19 genome. Between 10.85 and 11.16 % of the *D. buzzatii* st-1 genome is made up of TEs, between 7 and 7,5 % of *D. buzzatii* j-19 genome, while TEs represent 15.35 % of the D. mojavensis genome. Helitrons are the most abundant order in the three genomes.

**Conclusions:**

TEs in *D. buzzatii* are less abundant than in *D. mojavensis*, as expected according to the genome size and TE content positive correlation. However, TEs alone do not explain the genome size difference. TEs accumulate in the dot chromosomes and proximal regions of *D. buzzatii* and *D. mojavensis* chromosomes. We also report a significantly higher TE density in *D. buzzatii* and *D. mojavensis* X chromosomes, which is not expected under the current models. Our easy-to-use correction method allowed us to identify recently active families in *D. buzzatii* st-1 belonging to the LTR-retrotransposon superfamily Gypsy.

**Electronic supplementary material:**

The online version of this article (doi:10.1186/s12864-016-2648-8) contains supplementary material, which is available to authorized users.

## Background

Transposable elements (TEs) are mobile DNA sequences present in virtually all the eukaryote genomes sequenced and account for variable fractions of the genomes they inhabit. TEs are important not only because of their abundance but also because they are active components of the genomes, inducing structural rearrangements, inactivating or duplicating genes and adding or removing regulatory regions [1].

There are two classes of TEs, those that mobilize via an RNA intermediate belong to class I and those which transpose directly, leaving the donor site, or via a DNA intermediate, to class II [2, 3]. Further divisions in this classification comprise orders that distinguish TEs with different insertion mechanisms, and superfamilies that are composed of TEs with similar domain structures and protein sequences.

Progress in all aspects of genome sequencing and assembly has driven a revolution in the field. After *D. melanogaster* [4] and *D. pseudoobscura* [5] were sequenced, joint efforts provided the research community with the genomes of ten new Drosophila species which allowed multiple species comparisons [6]. These 12 genomes were sequenced with Sanger technology. After those, six *de novo* genomes were published individually [7–12], and eight more together [13]; these 14 genomes were sequenced mainly with Next-Generation Sequencing (NGS) technology.

The production of new genomes seems unstoppable and the comparisons and the knowledge drawn from them limitless. However, the information contained in some *de novo* draft genomes sequenced with NGS is not fully accurate [14, 15]. TEs, because of their repetitive nature, are at the root of most of the problems that cause misassemblies [16, 17]. Hence, contextualization and comparison of the TE fraction of genomes sequenced and annotated separately is difficult and scarce. The latest advances in sequencing technology [18, 19] and standardization in annotation methods [20] may contribute to solve this issue, but meanwhile, sequenced genomes keep piling up.

In this article, we analyze in detail the TE content of the *D. buzzatii* reference (st-1) genome [12], and compare it to that of a second *D. buzzatii* strain (j-19), described here, and that of *D. mojavensis*, another member of the *repleta* group [6]. We also compare the TE fraction in all available *Drosophila* genus genomes to test whether there are differences between NGS and Sanger-sequenced genomes, propose a method to correct such differences, and apply it to the genomes of two strains of *D. buzzatii*.

## Methods

### Genomes

The genomes used in this work were all freely available online except the genome of *D. buzzatii* strain j-19, which is described here and available through http://dbuz.uab.cat.

Strain j-19 was isolated from flies collected in Ticucho (Argentina) using the balanced-lethal stock Antp/ *Δ*^5^ [21]. Individuals of the j-19 strain are homozygous for the chromosome arrangement 2j [22]. DNA was extracted from male and female adults using the sodium dodecyl sulfate (SDS) method [23] or the method described by Piñol et al. [24] for isolating high molecular weight DNA. Three Illumina HiSeq Paired End (PE) libraries were prepared and sequenced at CNAG (Centro Nacional de Análisis Genómico) with an insert size of 500 bp and a mean read length of 102 bp. SOAPdenovo [25] version 1.05 was used to assemble the genome of the j-19 strain. We fed the assembler with 251,719,776 filtered reads setting the assembler with kmer size *k*=31. The final assembly contains 10529 scaffolds over 3 kb (total size = 153,440,896 bp). The N50 index is 1666, and the N50 length 24268 bp, the N90 index is 6825, and the N90 length 5747 bp.

Publicly available genomes from the *Drosophila* genus were downloaded from FlyBase (*D. ananassae* r1.3, *D. erecta* r1.3, *D. grimshawi* r1.3, *D. melanogaster* r6.05, *D. mojavensis* r1.3, *D. persimilis* r1.3, *D. pseudoobscura* r 3.2, *D. sechellia* r1.3, *D. simulans* r1.3 and r2.01 [26], *D. virilis* r1.2, *D. willistoni* r1.3, and *D. yakuba* r1.3 [6]), NCBI (*D. albomicans* [7], *D. biarmipes*, *D. bipectinata*, *D. elegans*, *D. eugracilis*, *D. ficusphila*, *D. kikkawai*, *D. miranda* [8], *D. rhopaloa*, *D. suzukii* [10], and *D. takahashii* [13]) or project web sites (*D. americana* H5 (http://cracs.fc.up.pt/~nf/dame/index.html) [11] and *D. buzzatii* st-1 (http://dbuz.uab.cat) [12]).

### Transposable element library

We built a custom library to annotate and classify the mobile elements in the *D. buzzatii* and *D. mojavensis* genomes. The library comprised already known repeats (FlyBase and Repbase) and *de novo* elements found in the *D. buzzatii* st-1 genome (RepeatModeler and Repclass). FlyBase’s canonical set of TEs (http://flybase.org/) were blasted [27] against an early assembly of the *D. buzzatii* st-1 genome. For each query, significant hits were manually inspected in order to recover the most complete copy. Repbase [28] repeats from *Insecta* species were added to the library. RepeatModeler (version 1.0.4) [29] was used with RepeatScout [30] and Recon [31] to identify repeats, and the RMBlast engine and Repbase database to classify them. Repclass [32] was used to classify repeats identified by RepeatScout. Elements classified by Repclass as being distinct from previously identified repeats, or as being more complete, were added to the library. Sequences classified as simple, satellite or low complexity repeats, were removed from the library. Additionally, a blast analysis was performed to filter non-TE related sequences. Sequences with significant hits (*e*-value blast < 1e-25) with *D. mojavensis* coding sequences (cds) and at the same time with no significant similarity to repeats deposited in Repbase were removed.

### Repeat annotation

To compare the three genomes of the two *Drosophila repleta* group species (*D. buzzatii* st-1, *D. buzzatii* j-19 and *D. mojavensis*), we masked them with RepeatMasker [33] (version 4.0.5) and RMBlast (version 2.2.27+) and the *D. buzzatii* custom library using the default options except for cut off (score value 250), nolow and norna. We used the RepeatMasker output files *.out to estimate the amount of nucleotides of each order and superfamily. We also used RepeatMasker, with cut off 250, nolow, and norna, to assess the TE content of the 27 available Drosophila genomes, from 25 species. To reduce library bias factor we used the RepBase *Insecta* library. The assembly size was used, in each case, to compute the percentage of transposable elements.

### Chromosomal analysis

We analyzed the TE distribution along the chromosomes of *D. buzzatii* st-1 and *D. mojavensis*. We used the previously mapped and oriented scaffolds, the 158 N90 scaffolds (145 Mb) of *D. buzzatii* [12], and the 11 N80 scaffolds (156 Mb) of *D. mojavensis* [34]. These scaffolds are the longest scaffolds that cover the 90 and 80 *%* of the entire assemblies of *D. buzzatii* st-1 and *D. mojavensis* respectively. Consequently, the shortest scaffolds which had not been mapped and are presumably the TE-richest could not be included in this analysis. The mapped scaffolds were broken down into 50 kb non-overlapping windows using bedtools (makewindows) and the TE nucleotides in each window were calculated using also bedtools (intersect). We plotted the TE density (TE bp/window length) for all windows, including those smaller than 50 kb from the tip of each scaffold, in the reported order.

To assess the TE-density in every chromosome, in the proximal regions and in the rest of the chromosome independently, another set of windows was made with the *D. buzzatii* and *D. mojavensis* mapped scaffolds previously mentioned. The most proximal 3 Mb of chromosomes X, 2, 3, 4 and 5 (∼10 *%* of the chromosome) were divided in 50 kb windows as well as the remaining ∼90 % of the chromosomes, and the entire chromosome 6. Only whole windows (50 kb) were taken into account. For each chromosome and region, we computed the mean TE-density and standard deviation and plotted the TE-density window distribution. Additionally, differences among these distributions (whole chromosome, proximal and central+distal regions) were tested with the two-sample Kolmogorov-Smirnov test.

### Correction

We mapped the reads used in the genome pre-assembly of *D. buzzatii* st-1 (21924977 reads from 454, Illumina, and Sanger) [12] with GS Reference Mapper (v2.9) (http://454.com/products/analysis-software) to the final *D. buzzatii* assembly using the default options. GS Reference Mapper aligned 95.3 *%* of the reads (20422434 reads), 20270 reads less than those used by gs-Assembler to build the pre-assembly. We also mapped the *D. buzzatii* j-19 Illumina reads to the *D. buzzatii* j-19 with Bowtie2. Every read base pair that mapped to a TE-annotated position was added up to calculate the coverage of the position. The corrected value for each TE order and superfamily is the sum of read base pairs annotated as part of that order or superfamily, divided by the average coverage. *D. buzzatii* st-1 average coverage is the genes average coverage, 22.37x, calculated with the same procedure used for the TEs, but with 13657 genes identified in *D. buzzatii* st-1 genome [12]. The average coverage for *D. buzzatii* j-19 is 160x, SOAPdenovo estimation.

## Results

### TE content in *D. buzzatii* and *D. mojavensis* assemblies

In *D. buzzatii* st-1, TEs account for 8.43 *%* of the assembly, about twice the value of TEs in *D. buzzatii* j-19 (4.15 *%*), but almost half of the value of *D. mojavensis* (15.35 *%*). In order to make a fair comparison, we also considered only 3-kb or longer scaffolds for *D. mojavensis*, 2419 (187.4 Mb) out of 6841 scaffolds (193.8 Mb). However, the TE fraction in *D. mojavensis* genome is still higher (14.35 *%*) than the fraction in both *D. buzzatii* strains. Henceforth, the complete *D. mojavensis* genome assembly was used for the subsequent analyses.

**Fig. 1:**
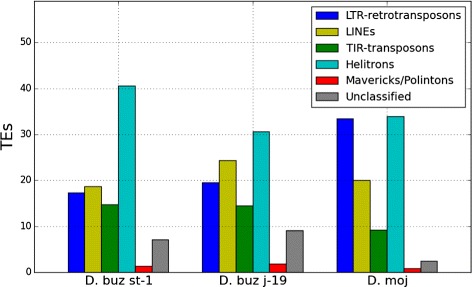
TE Order abundance. Percentage of transposable element orders relative to the mobile fraction of the genomes of *D. buzzatii* st-1, j-19, and *D. mojavensis*

The contribution of the different orders, defined by Wicker et al. [2], to the total amount of TEs (Fig. 1 and Table 1), is similar between the two *D. buzzatii* genomes (Helitrons, LINEs, LTR-retrotransposons, TIR-transposons, and Mavericks/Polintons), and differs from the *D. mojavensis* one. Despite the similarities, there are some differences. Although Helitrons are the most abundant order in the three genomes, they are more abundant in the *D. buzzatii* st-1 genome (40.61 *%* of the TEs content) than in the other two genomes (30.65 *%* in *D. buzzatii* j-19 and 33.90 *%* in *D. mojavensis*). LTR-retrotransposons are the second most abundant order in *D. mojavensis* (33.46 *%*), but not in *D. buzzatii* (17.38 *%* in st-1 and 19.54 *%* in j-19) where in both strains LINEs are the second most abundant order in genome contribution. TIR-transposons are more frequent in *D. buzzatii* genomes (14.81 *%* in st-1 and 14.46 *%* in j-19) than in *D. mojavensis* (9.24 *%*), like the unclassified repeats that are more abundant in *D. buzzatii* (7.15 *%* in st-1 and 9.11 *%* in j-19) than in *D. mojavensis* (2.42 *%*).

**Table 1:** TE contribution of every order and superfamily (kb) to the *D. buzzatii* (st-1 and j-19 before and after the correction) and *D. mojavensis* genomes^a^

Superfamily	*D. buz*				*D. moj*
	st-1	st-1 corr.	j-19	j-19 corr.	
	**2366.44**	**4693.31**	**1243.43**	**2050.57**	**9953.02**
**LTR Total**	**(17.38 %)**	**(26.03 %)**	**(19.54 %)**	**(17.62 %)**	**(33.46 %)**
BelPao	435.35	1025.76	198.65	432.82	2255.95
Copia	309.80	522.62	162.75	275.82	718.71
ERVK	10.92	9.97	8.09	7.52	18.06
Gypsy	1610.37	3134.95	873.94	1334.42	6960.30
	**2541.65**	**3401.72**	**1551.05**	**2221.12**	**5977.29**
**LINE Total**	**(18.66 %)**	**(18.87 %)**	**(24.37 %)**	**(19.08 %)**	**(20.09 %)**
CR1	396.35	761.48	117.39	546.88	947.96
I	74.63	136.15	20.19	38.59	110.53
Jockey	478.24	600.72	246.54	345.78	765.64
L1	6.71	6.01	6.70	5.63	8.08
L2	191.37	213.18	145.73	148.74	395.99
LOA	1.18	1.31	0.82	0.65	1.95
R1	1383.35	1663.22	1011.77	1133.23	3721.30
R2	1.49	9.30	0.51	0.38	23.03
R4	1.57	0.80	0.70	0.57	1.37
RTE	6.76	9.55	0.69	0.68	1.43
	**2016.98**	**2476.88**	**919.50**	**1820.64**	**2747.83**
**TIR Total**	**(14.81 %)**	**(13.74 %)**	**(14.46 %)**	**(15.64 %)**	**(9.24 %)**
hAT	563.03	661.13	239.06	414.90	654.13
Mutator	21.00	16.32	16.14	14.05	22.73
Novosib	17.35	16.43	11.89	10.77	16.15
P	590.70	830.17	216.28	713.43	752.39
PIF/Harbinger	3.81	9.71	2.21	2.45	7.82
piggyBack	18.67	9.46	5.38	5.79	77.21
Tc1/mariner	407.93	507.35	186.38	363.43	534.42
Transib	281.27	115.97	172.40	211.64	627.54
TIR other	113.23	310.35	69.75	84.18	55.43
	**5531.01**	**6331.89**	**1950.81**	**4689.50**	**10083.94**
**Helitron**	**(40.61 %)**	**(35.12 %)**	**(30.65 %)**	**(40.29 %)**	**(33.90 %)**
	**189.27**	**129.44**	**118.57**	**100.34**	**263.81**
**Maverick**	**(1.39 %)**	**(0.72 %)**	**(1.86 %)**	**(0.86 %)**	**(0.89 %)**
	**0.24**	**0.11**	**0.67**	**0.40**	**0.19**
**Others**	**(0 %)**	**(0 %)**	**(0 %)**	**(0 %)**	**(0 %)**
	**973.76**	**994.61**	**580.02**	**756.66c**	**721.26**
**Unknown**	**(7.15 %)**	**(5.52 %)**	**(9.11 %)**	**(6.50 %)**	**(2.42 %)**
**Total**	**13619.34**	**18027.96**	**6364.04**	**11639.23**	**29747.33**

^a^Order contributions, relative to the total TE fraction, are given in percentages

^b^Order total values are shown in boldface

### Chromosomal distribution

**Fig. 2:**
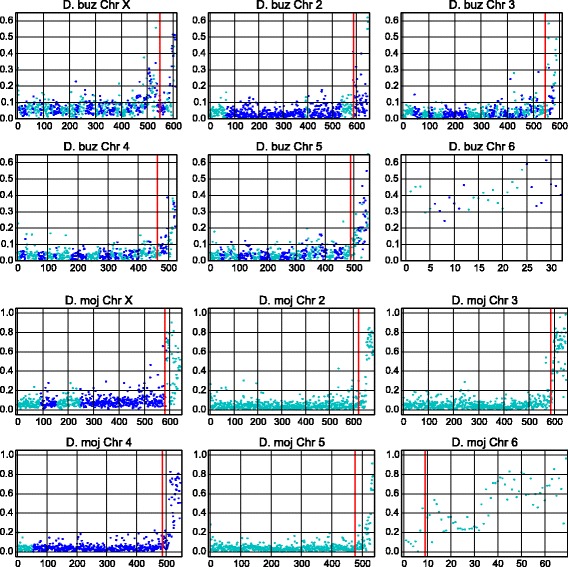
Chromosomal TE density. Density of transposable elements in 50 kb non-overlapping windows, starting (*left*) from the telomere. Only mapped and oriented scaffolds are included, N90 for *D. buzzatii* st-1, and N80 for *D. mojavensis*. Changes in *dot colors* denote scaffold changes and the *red lines mark* the most proximal 3 Mb of each chromosome

The TE distribution along *D. buzzatii* N90 mapped scaffolds and *D. mojavensis* N80 mapped scaffolds (Fig. 2) shows a similar pattern in both species: increased TE density in (i) chromosome 6 (the "dot" chromosome), (ii) the pericentromeric regions of all chromosomes, and (iii) chromosome X compared with the autosomes (Fig. 2). The density of the main orders plotted individually (Additional file 1: Figure S1a–h) reveals the prevalence of Helitrons in *D. buzzatii* proximal regions, specially the 3 Mb closest to the centromere.

We compared the abundance of TEs annotated in *D. buzzatii* and *D. mojavensis*, specifically the distribution of TE density in 50 kb windows, for whole chromosomes (the N90 mapped scaffolds of *D. buzzatii* and the N80 mapped scaffolds of *D. mojavensis*), for proximal regions (3 Mb), and for central and distal regions (Table 2). It is important to note that only the largest scaffolds are being considered, and that 10 and 20 *%* of *D. buzzatii* and *D. mojavensis* assemblies respectively, contained in the smallest and typically TE-enriched scaffolds, were discarded from this analysis. This explains the differences between the annotation of the whole assembly and the mean values of the mapped scaffolds. The smaller and TE-richer scaffolds are likely located in proximal regions, as the centromeric regions have the higher TE-density and more nested TEs. However, all recent TE insertions are susceptible to misassemblies and small scaffolds could be located between mapped scaffolds.

**Table 2:** TE fraction in *D. buzzatii* and *D. mojavensis* computed in 50 kb non-overlapping windows^a^

Chr	Species	Proximal		Cent+Dist		Total	
		TE (%)	N	TE (%)	N	TE (%)	N
X	*D. buzzatii*	16.13	57	7.44	505	8.32	562
	*D. mojavensis*	42.24	59	8.71	579	11.81	638
2	*D. buzzatii*	13.91	59	4.77	638	5.54	697
	*D. mojavensis*	38.68	60	5.11	622	8.06	682
3	*D. buzzatii*	12.96	58	4.12	522	5.01	580
	*D. mojavensis*	60.52	60	5.60	586	10.70	646
4	*D. buzzatii*	12.50	58	3.77	434	4.80	492
	*D. mojavensis*	39.24	60	4.31	486	8.14	546
5	*D. buzzatii*	14.98	58	4.06	462	5.87	520
	*D. mojavensis*	21.47	60	4.11	476	6.06	536
6	*D. buzzatii*	41.22	28	-	-	41.22	28
	*D. mojavensis*	50.65	60	14.22	8	46.30	68
Total	*D. buzzatii*	16.51	318	4.87	2561	5.86	2879
	*D. mojavensis*	42.13	359	5.68	2757	8.87	3116

^a^Proximal regions corresponds to the 3 most proximal Mb; Central+ Distal to the rest of the chromosome and Total to both parts. N stands for number of windows. Only mapped and oriented scaffolds are present, N90 for *D. buzzatii*, and N80 for *D. mojavensis*

*D. mojavensis* chromosomes, as a whole, or any of their parts, have a higher TE fraction than *D. buzzatii* chromosomes. The biggest differences are in the proximal regions, diminishing in the central and distal regions. Chromosome 6 (Muller element F) is the TE-richest chromosome in both species, 41.22 *%* in *D. buzzatii* and 46.30 *%* in *D. mojavensis*. In *D. buzzatii*, 8.32 % of chromosome X (Muller element A) is made up by TEs, followed by the other chromosomes with values between 4.80 and 5.86 *%*. In *D. mojavensis*, the X chromosome has 11.81 *%* of TEs, chromosome 310.70 *%* and the rest of the chromosomes have values between 8.14 and 6.06 *%*. *D. buzzatii* chromosomes 6 and X, when analyzed as a whole, are the only ones with TE density distributions significantly different (two-sample Kolmogorov-Smirnov test *p* < 0.001) from all other chromosomes, whereas in *D. mojavensis* it is chromosomes 6, X, and 3 (Additional file 2: Tables S1, S2, S3 and S4) that show significant differences. If we discard the 3 most proximal Mb and chromosome 6, chromosome X of both species is the only one with significantly different TE density distribution from all the other chromosomes (Additional file 2: Tables S5, S6, S7 and S8). When the pericentromeric regions are compared, in *D. buzzatii* there are not significant differences among chromosomes, while among *D. mojavensis* proximal regions, chromosome 3 TE density is significantly different from the rest of the chromosomes (Additional file 2: Tables S9, S10, S11 and S12). Consequently, in both species, chromosomes 6 and X display a significantly different TE distribution pattern from the rest of the chromosomes.

### Impact of the sequencing method in *Drosophila* genus

Because the genomes of *D. mojavensis*, *D. buzzatii* st-1 and j-19 strains were sequenced with different platforms and assembly strategies (see Methods), the differences in TE content between these genomes could be related to the methodologies used. More specifically, the Sanger sequenced *D. mojavensis* genome [6] shows a higher TE content than the *D. buzzatii* reference (st-1) genome sequenced with 454, Illumina and Sanger [12], which itself has a higher TE content than the *D. buzzatii* j-19 genome sequenced only with Illumina. Therefore it seems that NGS yields a smaller repeat content than Sanger sequencing [35].

**Fig. 3:**
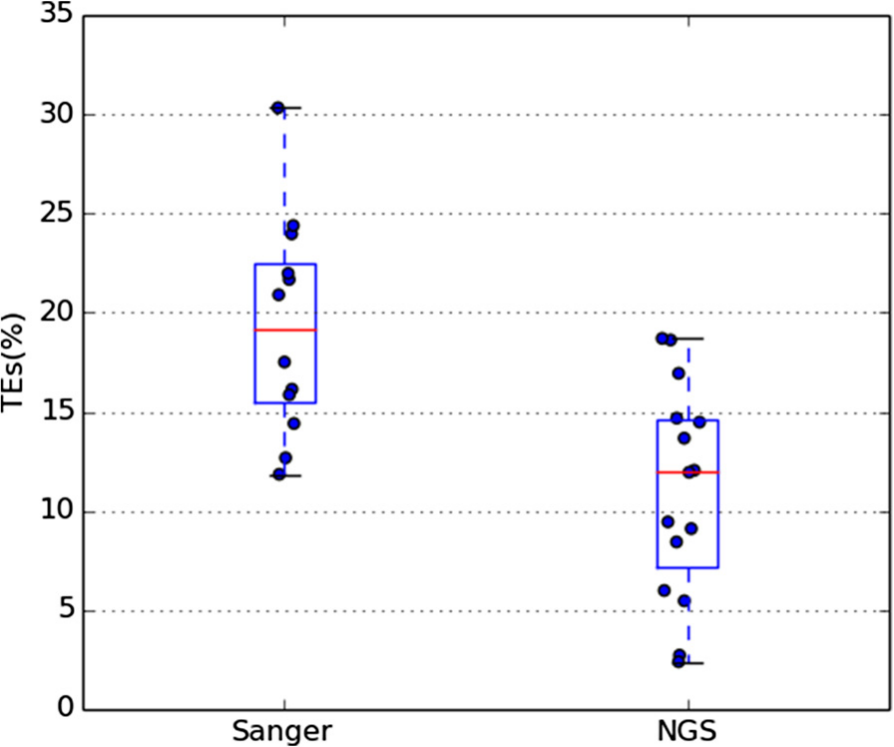
TEs in Sanger and NGS genomes. Boxplot representing the TE *%* in Drosophila genomes

In order to test this hypothesis, we widened our scope to include all the available genomes of *Drosophila* genus (Table 3). As in the cases of *D. mojavensis* and *D. buzzatii* there is a difference in the mobile fraction depending on the sequencing method. The mean TE percentage in the 12 genomes sequenced with Sanger technology is 19.31 *%*, whereas that in the 15 newly sequenced genomes (chiefly produced using NGS) is 10.98 *%*. The differences are significant (Mann-Whitney U-test *p*-value = 0.001421) and clear when the values are plotted (Fig. 3).

**Table 3:** Percentage of TEs annotated with repeat masker and RepBase *Insecta* library on every available genomes of *Drosophila* genus

Species	Subgenus	Group	Subgroup	Seq method	TEs
*D. albomicans*	Drosophila	immigrans	nasuta	NGS	2.73
*D. buzzatii* st-1	Drosophila	repleta	mulleri	NGS	5.99
*D. buzzatii* j-19	Drosophila	repleta	mulleri	NGS	2.40
*D. mojavensis*	Drosophila	repleta	mulleri	Sanger	16.14
*D. americana*	Drosophila	virilis	virilis	NGS	9.11
*D. virilis*	Drosophila	virilis	virilis	Sanger	17.51
*D. grimshawi*	Hawaian	grimshawi	grimshawi	Sanger	15.86
*D. ananassae*	Sophophora	melanogaster	ananassae	Sanger	30.33
*D. bipectinata*	Sophophora	melanogaster	ananassae	NGS	16.94
*D. elegans*	Sophophora	melanogaster	elegans	NGS	12.05
*D. eugracilis*	Sophophora	melanogaster	eugracilis	NGS	13.67
*D. ficusphila*	Sophophora	melanogaster	ficusphila	NGS	9.45
*D. erecta*	Sophophora	melanogaster	melanogaster	Sanger	14.41
*D. melanogaster*	Sophophora	melanogaster	melanogaster	Sanger	21.67
*D. sechellia*	Sophophora	melanogaster	melanogaster	Sanger	20.90
*D. simulans*	Sophophora	melanogaster	melanogaster	Sanger	11.85
*D. simulans*	Sophophora	melanogaster	melanogaster	NGS	8.44
*D. yakuba*	Sophophora	melanogaster	melanogaster	Sanger	21.98
*D. kikkawai*	Sophophora	melanogaster	montium	NGS	11.95
*D. rhopaloa*	Sophophora	melanogaster	rhopaloa	NGS	18.62
*D. biarmipes*	Sophophora	melanogaster	suzukii	NGS	14.48
*D. suzukii*	Sophophora	melanogaster	suzukii	NGS	18.70
*D. takahashii*	Sophophora	melanogaster	takahashii	NGS	14.68
*D. miranda*	Sophophora	obscura	obscura	NGS	5.47
*D. persimilis*	Sophophora	obscura	obscura	Sanger	23.97
*D. pseudoobscura*	Sophophora	obscura	obscura	Sanger	12.68
*D. willistoni*	Sophophora	willistoni	willistoni	Sanger	24.39

It is possible that the species sequenced with Sanger technology have *per se* more TEs than those sequenced with NGS, and sequencing or assembly methods do not influence the assembly TE fraction. However, when species belonging to the same subgroup are compared, the Sanger-sequenced genomes show a consistently higher percentage of TEs. The *mulleri* subgroup species, *D. buzzatii* and *D. mojavensis*, have different values than those yielded by our custom library but the pattern is the same. More examples (Table 3) are in the *virilis*, the *ananassae* or the *obscura* subgroups, where the species sequenced with shorter reads have a lower percentages of mobile elements. Two genomes from the *virilis* subgroup have been sequenced, *D. virilis* with Sanger and *D. americana* with NGS, and have 17.51 and 9.11 *%* of TEs respectively. *D. ananassae* sequenced with Sanger has 30.33 *%* of TEs, *D. bipectinata* sequenced with NGS has 16.94 *%*. Similarly, *D. persimilis* and *D. pseudoobscura*, sequenced with Sanger technology, have 23.91 and 12.68 *%* respectively, whereas *D. miranda*, sequenced with NGS, has 5.47 *%* of TEs in its genome. Moreover, the case of the same species sequenced by both technologies further supports the trend. *D. simulans* has been recently resequenced with NGS and old Sanger sequences to amend significant problems with the previous Sanger project. Our results show that the newly sequenced genome has 8.44 *%* of TEs (6.85 *%* according to Hu et al. [26], the authors of the latter assembly) while the old assembly has 11.85 *%*. Although various methodologies of repeat detection render various results, the use of the same procedure on Sanger and primarily NGS genomes gives consistently higher values of repeats in Sanger genomes. Hence, to accurately compare the results of *D. buzzatii* genome to other Sanger genomes like *D. mojavensis*, we thought it was necessary to correct our previous estimates of the *D. buzzatii* TE fraction.

### Correction of TE estimation by coverage

We found 403.3 Mb of reads, out of 3609 Mb, mapping to regions annotated as TEs in *D. buzzatii* st-1 assembly, corresponding to 11.16 *%* of all reads mapped. After dividing this 403.3 Mb by the average gene coverage (22.37 ×) we got the corrected value of TEs of *D. buzzatii*, 18 Mb. Therefore there is a 1.32 fold underestimation (4.4 Mb) with respect to the 13.6 Mb initially annotated with RepeatMasker. If we keep considering the assembly size as the genome size, and assume the extra 4.4 Mb belong to the gaps within scaffolds (15 Mb) the initial estimate of TEs in the genome of 8.43 *%* increases to 11.16 *%*. On the other hand, if we add the 4.4 new Mb to the assembly size, we get a genome size of 165.9 Mb and the TE fraction is 10.85 *%*. The correction, also applyed to *D. buzzatii* j-19 genome, revealed that TEs correspond to 11.64 Mb instead of the 6.4 Mb annotated, that means an increase from 4.15 to 7.59 *%* (7.05 *%* if we add the new 6.4 Mb to the genome size). We conclude that the TE fraction in *D. buzzatii* st-1 is between 10.85 and 11.16 *%* and between 7.59 and 7.05 *%* in *D. buzzatii* j-19.

**Fig. 4:**
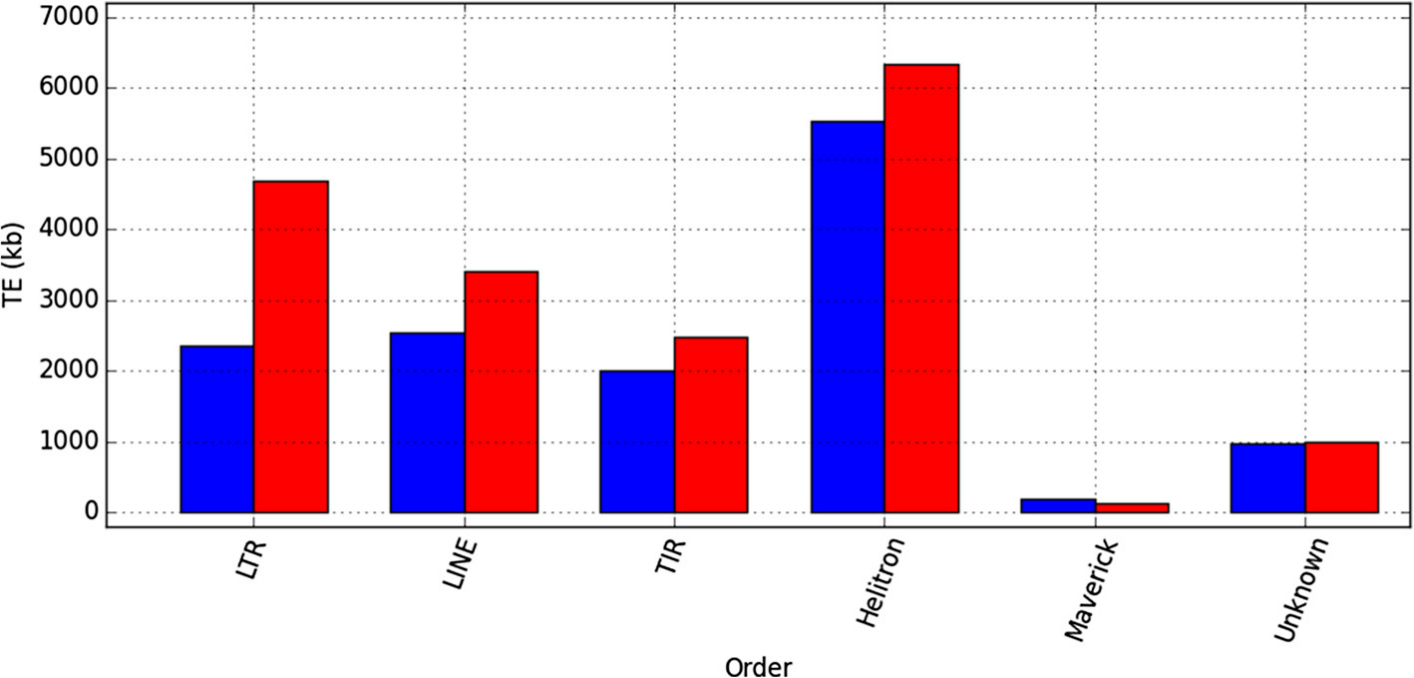
Order correction. Main order contribution (kb) to *D. buzzatii* st-1 genome, before (*blue*) and after (*red*) the coverage-based correction

**Fig. 5:**
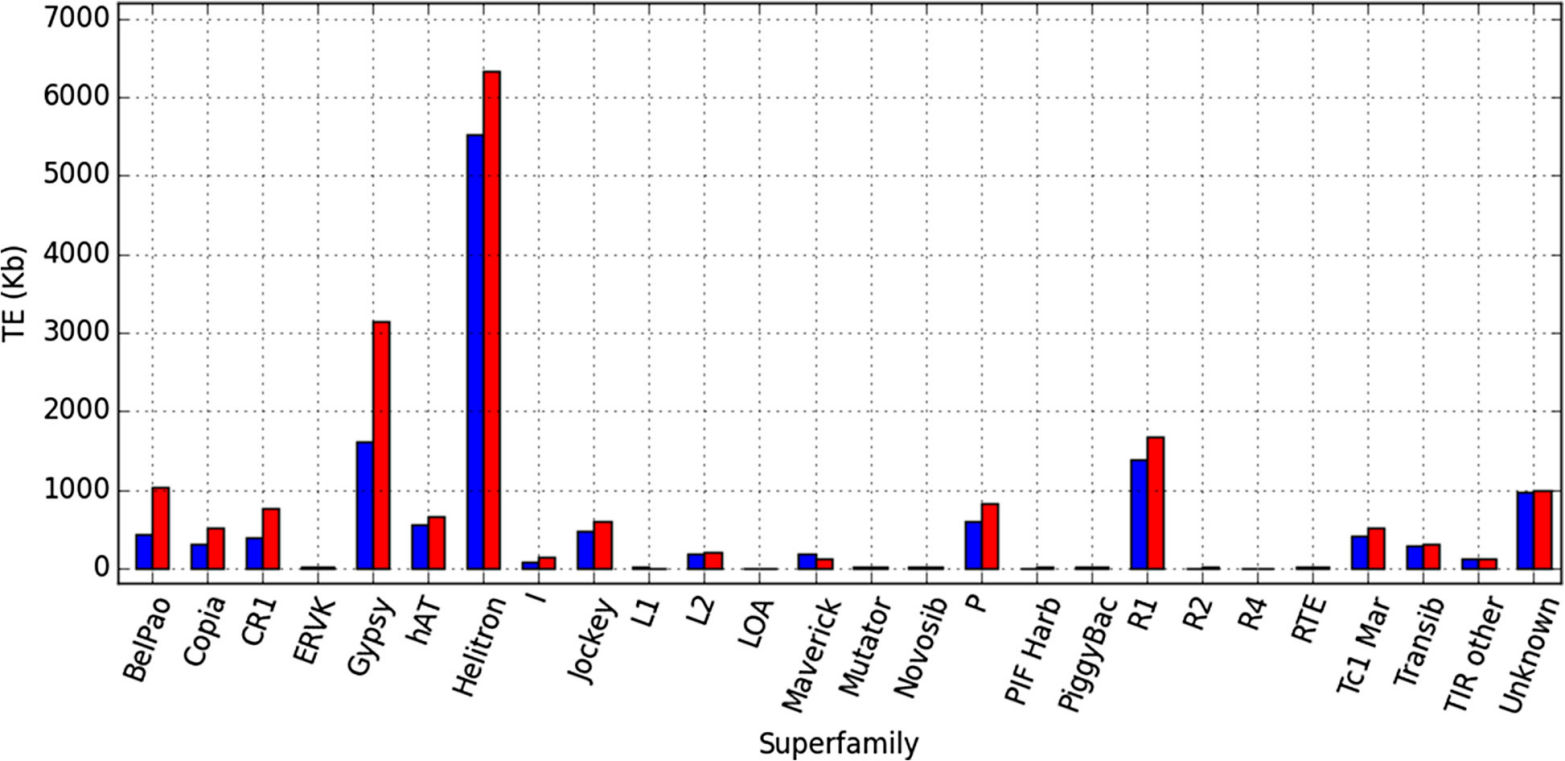
Superfamily correction. Superfamily contribution (kb) to *D. buzzatii* st-1 genome before (*blue*) and after (*red*) the coverage-based correction

Consequently, the orders and superfamilies with a higher correction factor are the ones with copies missing in the assembly. The results (Fig. 4 and Table 1) show that LTR-retrotransposons are the most underestimated order in *D. buzzatii* st-1 annotation by a factor of 1.98. At the superfamily level (Fig. 5), Gypsy and BelPao are the most underestimated in *D. buzzatii* st-1 annotation, increasing after the correction by more than two fold.

*D. buzzatii* st-1 and *D. mojavensis* TE profiles are more similar to each other after the correction as *D. buzzatii* LTR-retrotransposons have now overtaken LINEs as the second most frequent order. LINEs are underrepresented in the genome annotation by a factor of 1.34. The superfamilies CR1 and R1 increase by 365 and 280 kb respectively after the correction. The R2 superfamily represents a singular case, since it is not relevant in absolute value (1.5 kb annotated), but the correction factor is the highest of all superfamilies (6.24 fold) and, after the correction, 9.3 kb are found to belong to the R2 superfamily. TIR-transposons are underestimated in the annotation by a 1.23 factor, with most superfamilies having a fair representation (correction factor close to one), but due to its large size, this small factor correction represent a substantial change in the base count. After the correction, the P superfamily sequence increased by 239 kb (1.41 fold), Tc1/mariner cover 99 new kb (1.24 fold) and hAT 98 kb (1.17 fold). Helitrons are underestimated by a 1.15 factor, but like TIR-transposons, their abundance in the genome prior to the correction (5.5 annotated Mb) translates into a remarkable increase, 800 kb absent from the annotation. The correction, applyied to *D. buzzatii* j-19 reveals that Helitrons are heavily underrepresented in the annotation, while the LTR-retrotransposons are not as underestimated as in *D. buzzatii* st-1 (Table 1 and Additional file 1: Figures S2 and S3). Among superfamilies P, Helitron, and BelPao are the more underestimated in *D. buzzatii* j-19 assembly, by 3.3, 2.4 and 2.18 factors respectively. Gypsy superfamily is also remarkable if we look at the amount of new sequences with 460 new Kb. These superfamilies are likely to include highly similar insertions probably recently transposed.

## Discussion and conclusions

We have shown that *D. buzzatii* st-1 and j-19 genomes have a lower TE percentage than *D. mojavensis*. We have also reported that there is an underestimation of the mobile fraction of genomes sequenced with Next Generation Sequencing, possibly due to sequencing and assembly methods, which affect *D. buzzatii* st-1 genome, and j-19.

We have proposed a method based on read coverage to assess the magnitude of the bias, and used it to correct the *D. buzzatii* st-1 and j-19 TE estimates. In *D. buzzatii* st-1 the correction revealed another 4.4 Mb of TEs and increased the TE percentage to 11 *%*, while for *D. buzzatii* j-19 five new Mb of TEs were found, meaning TEs are 7 *%* of the genome. Thus, although the TE content in *D. buzzatii* genome increased with the correction, it is still lower than that of *D. mojavensis* genome. Our methodology does not allow us to locate the TEs absent from the assembly. However, we consider it is important to describe the TEs present in the published assembly for several reasons. The differences while affecting particularly some orders and superfamilies have a small effect in others. Moreover, *D. buzzatii* uncorrected TE chromosomal distribution shows the same trends than those we observed in *D. mojavensis*. Finally, the published assembly should be analyzed and its limitations assessed in order to become a useful resource.

### *D. buzzatii* and *D. mojavensis* assembly TE content

Our results show that TEs in *D. buzzatii* genome are less abundant than in *D. mojavensis* genome, even after taking into account the bias correction. The size of the two genomes have been estimated by Feulgen Image Analysis Densitometry and the *D. buzzatii* genome estimates are between 21 *%* (st-1) and 25 *%* (j-19) smaller than those for *D. mojavensis*. Thus, our results agree with the well known positive correlation between genome size and transposable element fraction [36–38]. However, the difference in TE content does not explain the difference in size between the two genomes. Interestingly, after the coverage-based correction applied to *D. buzzatii* st-1, the contribution of each order to the total TE content is more similar to that of *D. mojavensis*, suggesting that the changes that lead to the differences affected every order in a uniform manner.

There are several non-mutually excluding explanations for the wide diversity in genome sizes and the forces driving its variation. The mutational explanation, ascribe part of such diversity to differences in insertion and deletion rates among species [39, 40]; other authors suggest that non-adaptative forces have diminished the efficiency of selection, explaining genome expansions [41]; positive natural selection proposes that genome size constraints may be different depending of the lineage history [42]. According to Charlesworth and Barton [42], having a larger genome size may be advantageous, or at least not as strongly selected against, in some scenarios. Genome size has been reported to be negatively correlated with developmental rate, which is also negatively correlated with body size [43, 44]. Hence, species without a constrain on developmental time and favored by a larger body size may have accumulated more repetitive sequences than closer species with developmental time constraints.

This is possibly the case of *D. buzzatii*, which generally lay its eggs in rotting tissues of several Opuntia cacti, although it can occasionally use columnar cacti [45–47]; while *D. mojavensis* primarily uses larger rotting columnar or barrel cacti (*Stenocereus gummosus* and *Stenocereus thurberi*, and *Ferocactus cylindraceous*), except for the Santa Catalina Island population that uses Opuntia [48–51]. In other words, *D. buzzatii* individuals mainly live in smaller cacti which dry faster, consequently a more ephemeral resource than those used by *D. mojavensis*. The selective pressure to keep a faster development in *D. buzzatii*, or the relaxation of this pressure in *D. mojavensis* could be behind their different genome size and TE contribution.

### Chromosomal distribution of TEs

TEs in *D. melanogaster* have been reported to accumulate in the proximal regions of the chromosomes, the transition between euchromatin and heterochromatin, where the recombination rate drops. The dot chromosome, which has a recombination rate considered null [52], has the highest TE density of all chromosomes [53, 54]. Moreover, recent analyses of several *D. melanogaster* populations have found a negative correlation between recombination rate and TE population frequency [55, 56].

TE dynamics has been extensively studied; however there is not a consensus about why some regions have a higher TE density. Ectopic recombination is so far the only explanation for the negative correlation between recombination rate and TE frequency. Recombination events involving non-homologous TE copies can lead to chromosomal rearrangements and inviable gametes [57]. According to the ectopic recombination hypothesis, the decrease in the recombination rates, seen in centromeric and telomeric regions, weakens the selection against TE insertions by reducing the crossing-over events between non-homologous TE copies [52, 58]. Accumulation of specific transposable elements in *D. buzzatii* centromeric regions was previously noticed using in situ hybridization [59, 60]. Additionally *D. mojavensis* dot chromosome TE density has also been found to be higher than that of *D. melanogaster*, *D. erecta* and *D. grimshawi* [61]. We are now reporting TE accumulations in the dot chromosomes and in the proximal regions of the rest of the chromosomes of *D. buzzatii* st-1 and *D. mojavensis*. The available linkage maps for *D. buzzatii* and *D. mojavensis* [62, 63] are not very detailed; even so, we can assume that like in *D. melanogaster* these regions have a reduced recombination rate.

The X chromosome poses a challenge when trying to explain its TE dynamics. Because the X has a higher recombination rate than the autosomes, and mutations are directly exposed to selection in hemizygous males, deleterious insertions should be removed more efficiently in the X chromosome than in the autosomes. An early analysis of the *D. melanogaster* reference genome showed a reduced accumulation of TEs in the *D. melanogaster* X chromosome [64]. However, recent analyses have surveyed several *D. melanogaster* populations and have not found evidence of a lower TE presence in the X chromosome, and some have even reported a higher abundance [55, 56, 65]. Our observations show that in *D. buzzatii* and *D. mojavensis* the X chromosome has a significantly higher TE density than the autosomes, except for the dot. And this difference remains even when the most proximal 3 Mb are discarded. Interestingly, the increase is sustained throughout the whole length of chromosome X in both species (Fig. 2). The X higher TE density is observed not only in *D. buzzatii* but also in *D. mojavensis*. Consequently, the assembly problem, that could have more impact on chromosome X as using males and female flies implies a lower coverage, does not seem to explain our results. The argument that some families with an insertion preference for the X have recently suffered an expansion in *D. melanogaster* [65] is interesting and may suggest that *D. buzzatii* and *D. mojavensis* TEs are actively transposing. However, there are possibly other factors, besides recombination, needed to understand the unpredicted TE abundance in the X chromosome.

### TEs and NGS

Issues with the NGS genomes repeats have been reported before [35] suggesting that stringent assembly strategies and shorter reads do not produce an accurate representation of the repeats in a specific *locus* but a consensus built with sequences from other *loci* [66]. Hence, the differences found in TE content between Sanger and NGS genomes are likely caused by an underestimation of NGS assembly methods rather than by an overestimation of TEs by Sanger technology. Although dealing with different technologies, it resembles the case of *D. melanogaster* Release 3 [67], where after extensive experimental efforts, most of the repetitive sequences of the previous release were found to be composite sequences of the newly sequenced TEs. It is also important to note that Sanger genomes, assembled with longer reads, may recover a longer fraction of the heterochromatin and go deeper in this region rich in repeated sequences than genomes sequenced with NGS. Consequently, comparing the mobile fraction of the two strains of *D. buzzatii* between them (st-1 sequenced with a mixture of Sanger, Illumina and 454 reads and j-19 sequenced solely with Illumina reads) and to *D. mojavensis* genome (sequenced with Sanger reads) raised questions about the reliability of such comparisons.

To find out if the sequencing technology, and potentially the assembly methods, implied major differences in TE annotation, we look at published genomes and their analyses of TE fractions. Two dozens of genomes of different *Drosophila* genus species have been released since *D. melanogaster* reference genome. Nevertheless, the mobile fraction of most of the recently published genomes has not been analyzed or has only been analyzed superficially [7–9, 11] yet there are some exceptions [10]. At least two analyses comparing some of these genomes in a uniform manner have been published [6, 9] but they yielded very different values. The main reasons seem to be the use of different annotation methods and updates in the TE libraries. The discrepancies between estimations compelled us to analyze all the *Drosophila* genus genomes available simultaneously, in the most homogeneous way possible and trying to reduce the unavoidable bias of library specificity. The values differ from previous studies but the comparisons should be more consistent. We found that genomes sequenced with Sanger technology have a higher TE percentage than those sequenced mainly with Illumina and 454 technologies. Because the data is not phylogenetically independent it is possible that species sequenced with one technology have actually a higher TE fraction than the ones sequenced with the other. However, from all the species from the same subgroup, sequenced with different technologies, the ones sequenced with Sanger show the highest TE percentage, suggesting that there is indeed an impact from the sequencing technology.

### Correction of *D. buzzatii* TE estimates

We mapped the reads used in the *D. buzzatii* assembly back to the assembly, following the lead of several projects that used high quality reference genomes and re-sequenced data from different individuals to accurately identify TE insertions [55, 56, 68, 69]. The mapping showed how some regions annotated as TE insertions had a TE coverage depth much higher than the surrounding regions. We also noticed that some gaps had TE annotations from the same family on each side, suggesting that the gap should be filled with TE sequence. In order to obtain a reliable estimate and account for the problems related to NGS (see above), we directly counted how many read nucleotides belonged to TEs. One could argue that some of those reads may belong to the heterochromatin, were casted aside during the assembly, and have been aligned now to euchromatin repeats. However, in *D. buzzatii* st-1 correction GS Reference Mapper aligned 20270 reads less in this process than those used by GS Reference Assembler. After mapping and dividing by the average coverage, we pulled the data for every order and superfamily together.

Sequence similarity among TE family copies is related to its transpositional activity. TE families which have recently transposed will contain highly similar copies and will be the most affected by the assembly problems mentioned before. Therefore, our correction method is expected to have a higher impact on these families. Our results show that LTR-retrotransposons were the most affected order by *D. buzzatii* st-1 correction. Their recent activity and their double repetitive nature, as not only LTR-retrotransposon copies will generate similar reads, but the LTRs from a single copy can produce reads susceptible to be assembled together are likely explanations. Additionally, LTR-retrotransposons are the longest TEs in Drosophila genomes, thus suffering more than other orders the artificial fragmentation by identification software [32] and assembly problems due to reads that do not span the lenght of the insertions. *Osvaldo* and *Isis* elements, from the Gypsy superfamily, were reported to be active in *D. buzzatii* [70, 71], which agrees with our ours results as Gypsy is the LTR-retrotransposon superfamily with a higher correction rate for *D. buzzatii* st-1 and also a high rate for *D. buzzatii* j-19. The LINEs superfamilies R1 and R2 are nested within ribosomal regions, typically poorly assembled, explaining their underestimation in *D. buzzatii* st-1 genome [72, 73].

*D. melanogaster* genome annotations and analyses of only euchromatic and both euchromatic and heterochromatic regions find the same order in the abundance of the major TE orders. According to [53, 74, 75] the contribution order is, from highest to lowest, LTR retrotransposons, LINE elements, TIR transposons, and Helitrons (when DINE-1 is annotated). This same order was found for most species in [6] work. However, it appears to be a difference in *Drosophila* subgenus order when Helitrons are taken into account. Yang and Barbash [76] carried out and extensive analysis of *DINE-1* on the firsts 12 Drosophila genomes sequenced. Their analyses revealed that *D. mojavensis* is the second in number of *DINE-1* copies, than those copies had probably undergone multiple rounds of transposition and silencing, and some had been recently transposed. Feschotte et al. [32] found that the *D. melanogaster* reported order was maintained in *D. pseudoobscura* and not in *D. virilis*, where Helitrons make up a higher fraction of the genome than TIR elements. This is in agreement with [9] observations for *D. virilis* and *D. mojavensis*, both from the *Drosophila* subgenus. Their analysis show how DNA elements, computing TIR elements and Helitrons together, are more abundant than LTR retrotransposons or LINE elements in these two species. Previous studies have already identified several families of Helitrons in *D. buzzatii* named ISBu (for Insertion Sequence of *D. buzzatii*) in chromosomal inversion breakpoints [77, 78]. We have now detected that over 800 kb of Helitrons were incorrectly assembled in *D. buzzatii* st-1, suggesting that 12.65 *%* of the Helitrons have been recently transposed, while 5531 kb of Helitrons are either sequenced in reads with other regions, that allowed the assembler to map them, or are not as similar to confound the assembler. Helitrons are also the most abundant order in *D. buzzatii* j-19 and is highly affected by the coverage-based correction. Hence, like in *D. mojavensis*, Helitrons seem to have undergone several rounds of activity and the TE content differences between *Drosophila* and *Sophophora* subgenera appear to be greater than initially thought.

Our methods has drawbacks; the correction does not inform of where the repeats are in the genome, or their specific sequence, an information that may not be precise in a NGS genome (see above). However, it is a method easy to apply that provides more acurate estimates of the abundance of each order and superfamily. Therefore, our strategy facilitates comparisons among the wealth of already sequenced genomes and deepens our understanding of genome evolution.

## Availability of data and material

The datasets supporting the conclusions of this article are available in the Drosophila buzzatii genome project repository http://dbuz.uab.cat and within the article additional files.

## Ethics approval and consent to participate

Not applicable.

## Consent for publication

Not applicable.

## Additional files

Additional file 1 Supplementary Figures. Supplementary Figure 1 (a to h). Chromosomal TE density. Main transposable element order density in 50 kb non-overlapping windows. Only mapped and oriented scaffolds are present, N90 scaffolds for *D. buzzatii* st-1 (a to d), and N80 scaffolds for *D. mojavensis* (e to h). Changes in dot colors denote scaffold changes. Supplementary Figure 2. *D. buzzatii* j-19 Order correction. Order contribution (kb) to *D. buzzatii* j-19 genome before (blue) and after (red) the coverage-based correction. Supplementary Figure 3. *D. buzzatii* j-19 Superfamily correction. Superfamily contribution (kb) to *D. buzzatii* j-19 genome before (blue) and after (red) the coverage-based correction. (ZIP 792 kb)

Additional file 2 Supplementary Tables. Supplementary Table 1. D statistics and *p*-values of U two-sample Kolmogorov-Smirnov tests comparing the distributions of TE densities in 50-Kb windows of each pair of chromosomes in three different sets: whole chromosome, central+distal and proximal regions. Only mapped and oriented scaffolds were considered. (PDF 389 kb)

## Abbreviations

BSC: Barcelona supercomputing center
Cds: coding sequences
CNAG: Centro Nacional de Aná1lisis Genómico
ISBu: insertion sequence of *D. buzzatii*
NGS: next-generation sequencing
PE: paired end
SDS: sodium dodecyl sulfate
TEs: transposable elements
