# Supplementary material for: Exploration of the Drosophila buzzatii transposable element content suggests underestimation of repeats in Drosophila genomes
Source: BMC Genomics. 2016 May 10;17:344. doi: 10.1186/s12864-016-2648-8 (PMC4862133; doi:10.1186/s12864-016-2648-8)
Supplement: Additional file 1 — Supplementary Figures. Supplementary Figure 1 (a to h). Chromosomal TE density. Main transposable element order density in 50 kb non-overlapping windows. Only mapped and oriented scaffolds are present, N90 scaffolds for D. buzzatii st-1 (a to d), and N80 scaffolds for D. mojavensis (e to h). Changes in dot colors denote scaffold changes. Supplementary Figure 2. D. buzzatii j-19 Order correction. Order contribution (kb) to D. buzzatii j-19 genome before (blue) and after (red) the coverage-based correction. Supplementary Figure 3. D. buzzatii j-19 Superfamily correction. Superfamily contribution (kb) to D. buzzatii j-19 genome before (blue) and after (red) the coverage-based correction. (ZIP 792 kb) [file 12864_2016_2648_MOESM1_ESM.zip › Supplementary_Figure_1d_Multiplot-dbuz_Helitron.pdf]

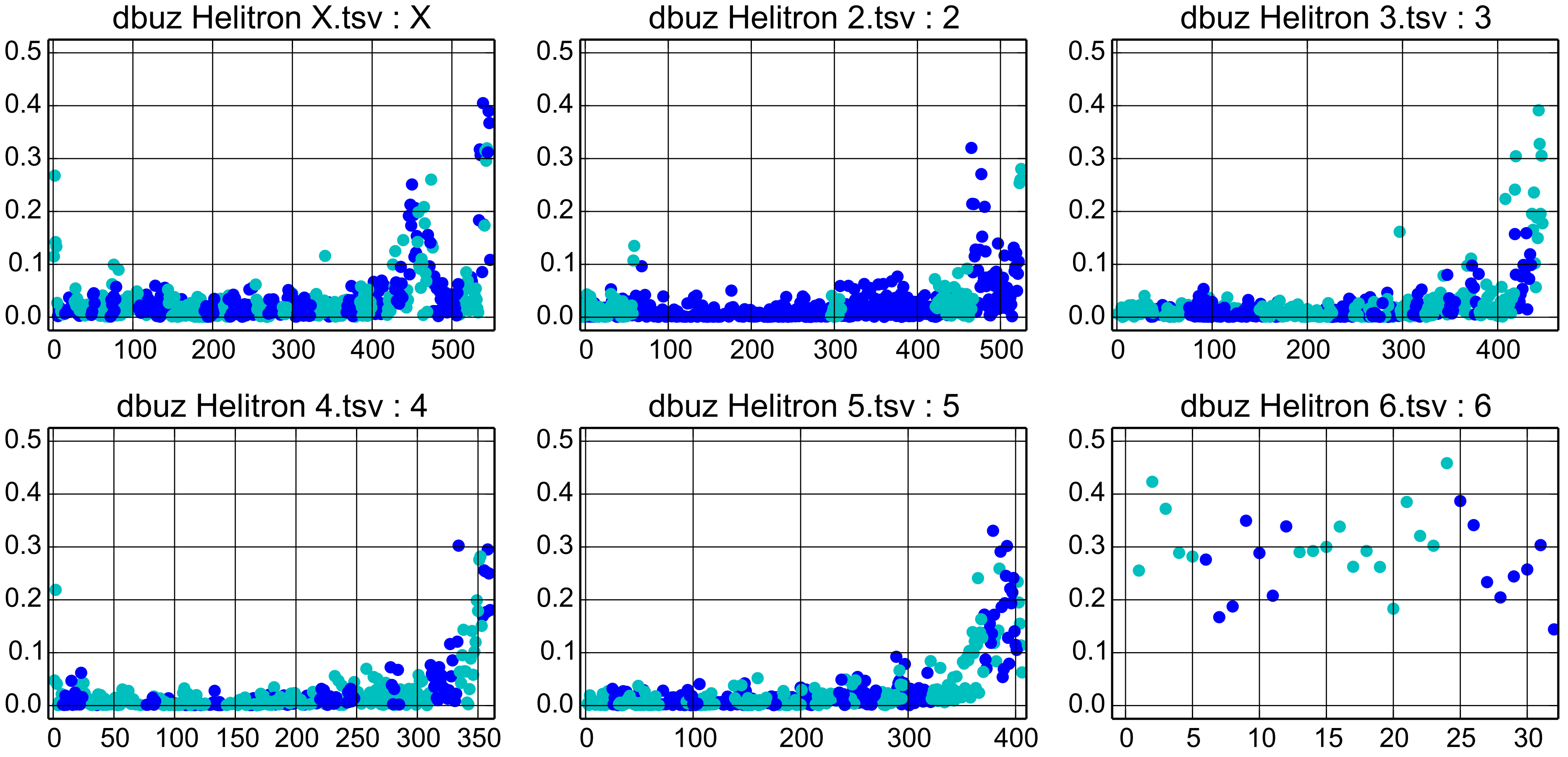
dbuz Helitron X.tsv : X
dbuz Helitron 2.tsv : 2
dbuz Helitron 3.tsv : 3
dbuz Helitron 4.tsv : 4
dbuz Helitron 5.tsv : 5
dbuz Helitron 6.tsv : 6
0.0 0.1 0.2 0.3 0.4 0.5
0 100 200 300 400 500
0 100 200 300 400
0 50 100 150 200 250 300 350
0 5 10 15 20 25 30
