# Supplementary figures and images for: Exploration of the Drosophila buzzatii transposable element content suggests underestimation of repeats in Drosophila genomes
Source: BMC Genomics. 2016 May 10;17:344. doi: 10.1186/s12864-016-2648-8 (PMC4862133; doi:10.1186/s12864-016-2648-8)

dmoj TIR X.tsv : X

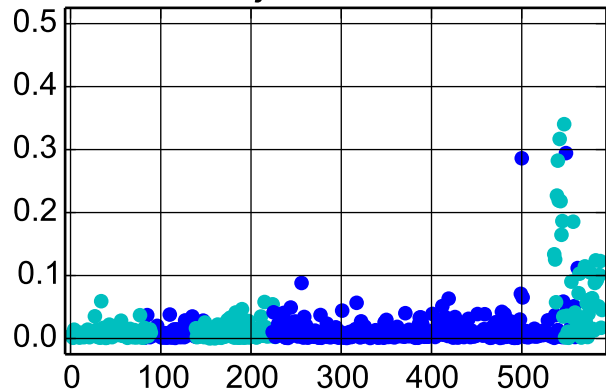

dmoj TIR 2.tsv : 2

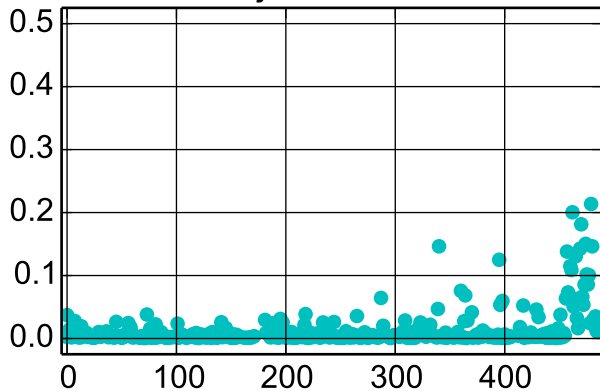

dmoj TIR 3.tsv : 3

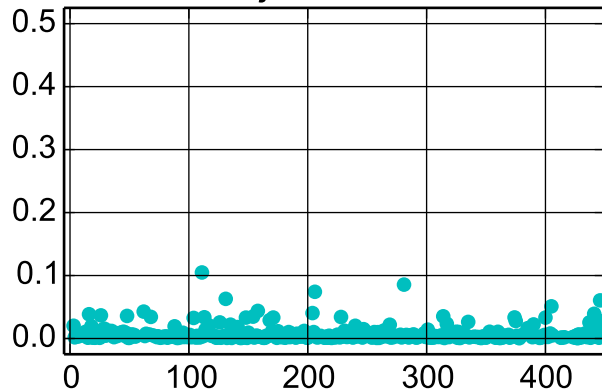

dmoj TIR 4.tsv : 4

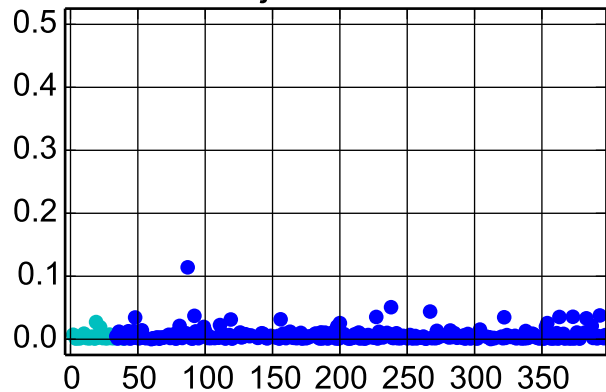

dmoj TIR 5.tsv : 5

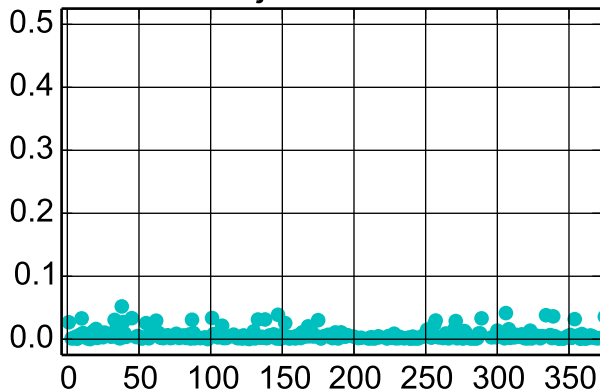

dmoj TIR 6.tsv : 6

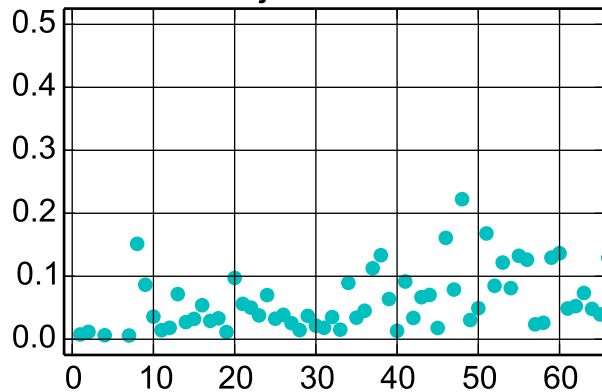

Supplement: Additional file 1 — Supplementary Figures. Supplementary Figure 1 (a to h). Chromosomal TE density. Main transposable element order density in 50 kb non-overlapping windows. Only mapped and oriented scaffolds are present, N90 scaffolds for D. buzzatii st-1 (a to d), and N80 scaffolds for D. mojavensis (e to h). Changes in dot colors denote scaffold changes. Supplementary Figure 2. D. buzzatii j-19 Order correction. Order contribution (kb) to D. buzzatii j-19 genome before (blue) and after (red) the coverage-based correction. Supplementary Figure 3. D. buzzatii j-19 Superfamily correction. Superfamily contribution (kb) to D. buzzatii j-19 genome before (blue) and after (red) the coverage-based correction. (ZIP 792 kb) [file 12864_2016_2648_MOESM1_ESM.zip › Supplementary_Figure_1g_Multiplot-dmoj_TIR.pdf]

dmoj LINE X.tsv : X

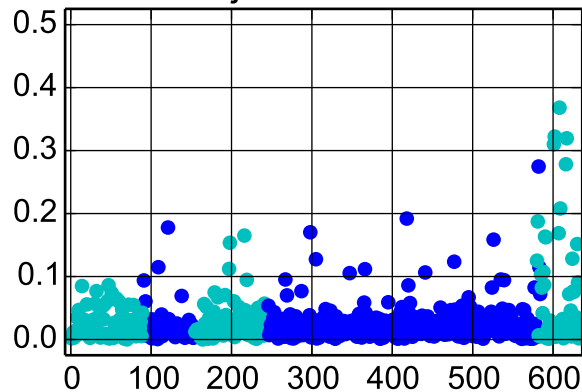

dmoj LINE 2.tsv : 2

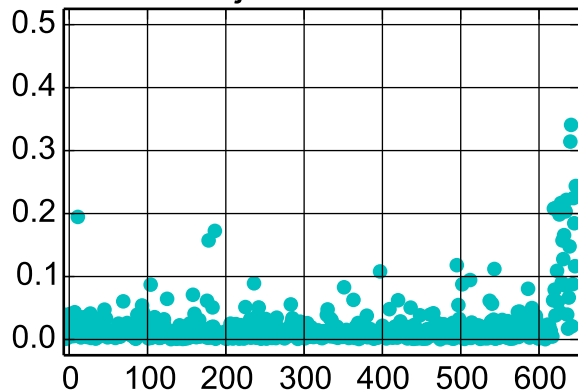

dmoj LINE 3.tsv : 3

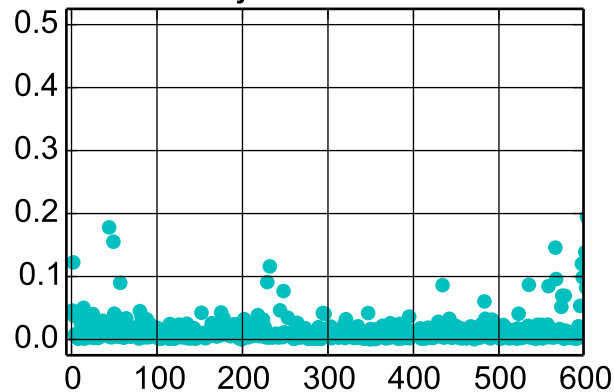

dmoj LINE 4.tsv : 4

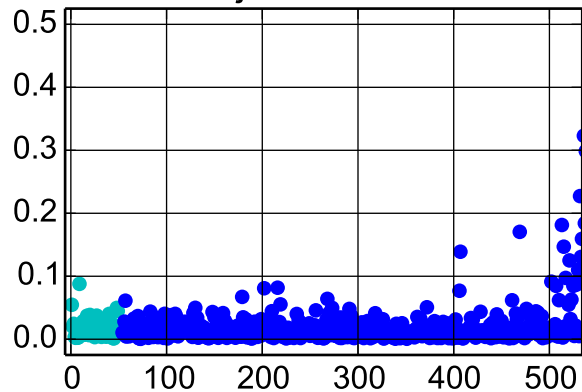

dmoj LINE 5.tsv : 5

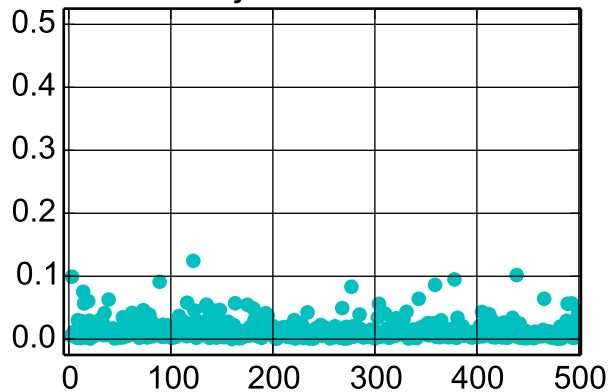

dmoj LINE 6.tsv : 6

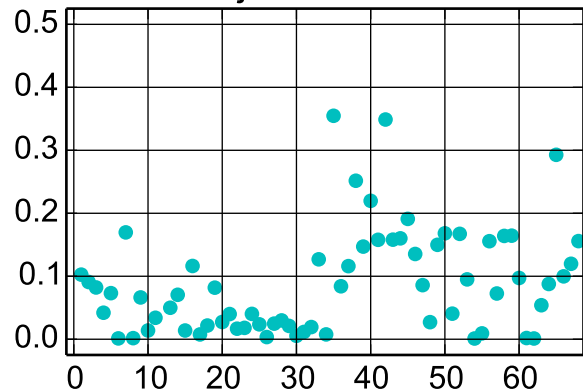

Supplement: Additional file 1 — Supplementary Figures. Supplementary Figure 1 (a to h). Chromosomal TE density. Main transposable element order density in 50 kb non-overlapping windows. Only mapped and oriented scaffolds are present, N90 scaffolds for D. buzzatii st-1 (a to d), and N80 scaffolds for D. mojavensis (e to h). Changes in dot colors denote scaffold changes. Supplementary Figure 2. D. buzzatii j-19 Order correction. Order contribution (kb) to D. buzzatii j-19 genome before (blue) and after (red) the coverage-based correction. Supplementary Figure 3. D. buzzatii j-19 Superfamily correction. Superfamily contribution (kb) to D. buzzatii j-19 genome before (blue) and after (red) the coverage-based correction. (ZIP 792 kb) [file 12864_2016_2648_MOESM1_ESM.zip › Supplementary_Figure_1f_Multiplot-dmoj_LINE.pdf]

dmoj LTR X.tsv : X

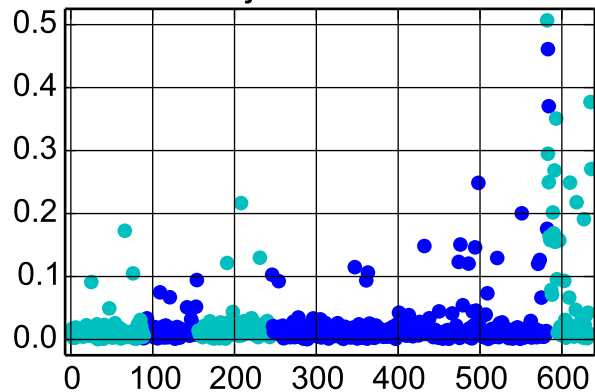

dmoj LTR 2.tsv : 2

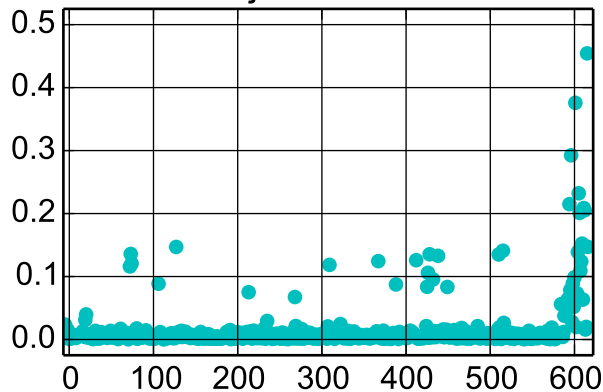

dmoj LTR 3.tsv : 3

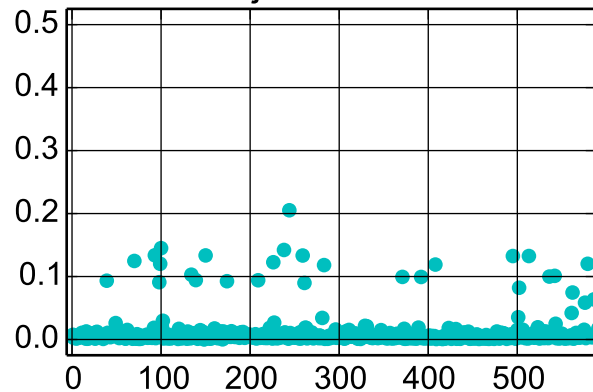

dmoj LTR 4.tsv : 4

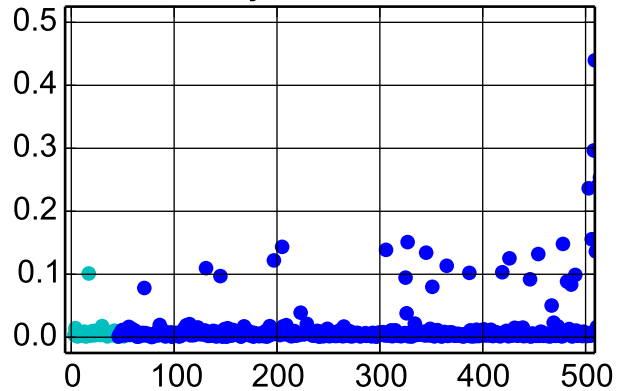

dmoj LTR 5.tsv : 5

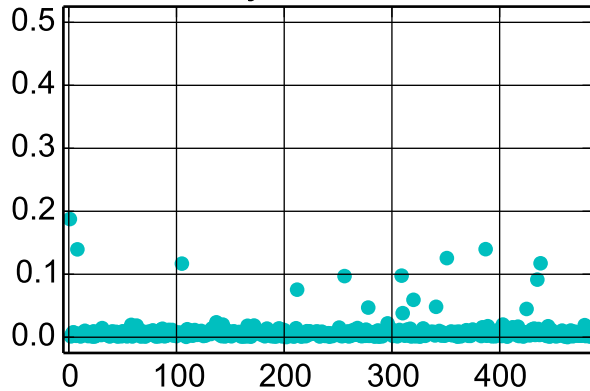

dmoj LTR 6.tsv : 6

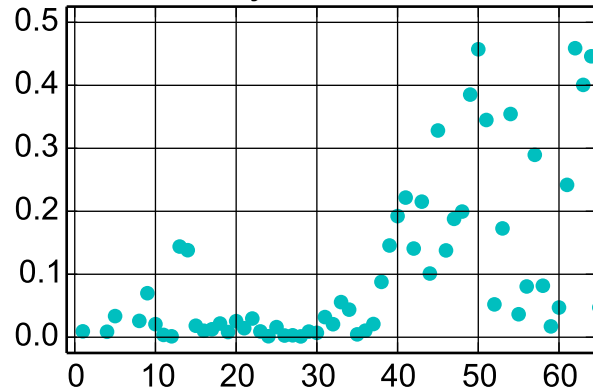

Supplement: Additional file 1 — Supplementary Figures. Supplementary Figure 1 (a to h). Chromosomal TE density. Main transposable element order density in 50 kb non-overlapping windows. Only mapped and oriented scaffolds are present, N90 scaffolds for D. buzzatii st-1 (a to d), and N80 scaffolds for D. mojavensis (e to h). Changes in dot colors denote scaffold changes. Supplementary Figure 2. D. buzzatii j-19 Order correction. Order contribution (kb) to D. buzzatii j-19 genome before (blue) and after (red) the coverage-based correction. Supplementary Figure 3. D. buzzatii j-19 Superfamily correction. Superfamily contribution (kb) to D. buzzatii j-19 genome before (blue) and after (red) the coverage-based correction. (ZIP 792 kb) [file 12864_2016_2648_MOESM1_ESM.zip › Supplementary_Figure_1e_Multiplot-dmoj_LTR.pdf]

dbuz TIR X.tsv : X

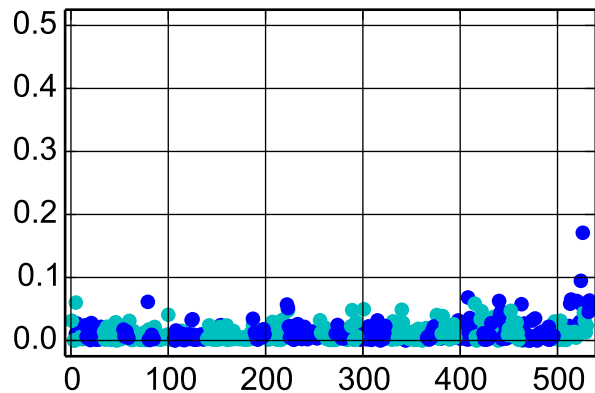

dbuz TIR 2.tsv : 2

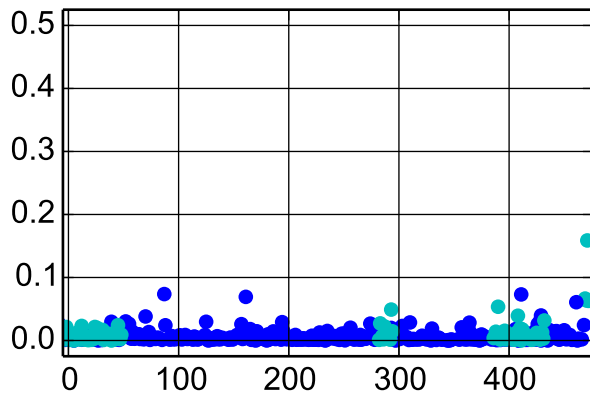

dbuz TIR 3.tsv : 3

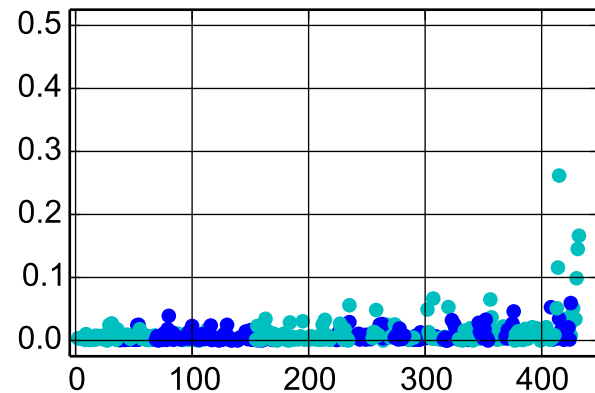

dbuz TIR 4.tsv : 4

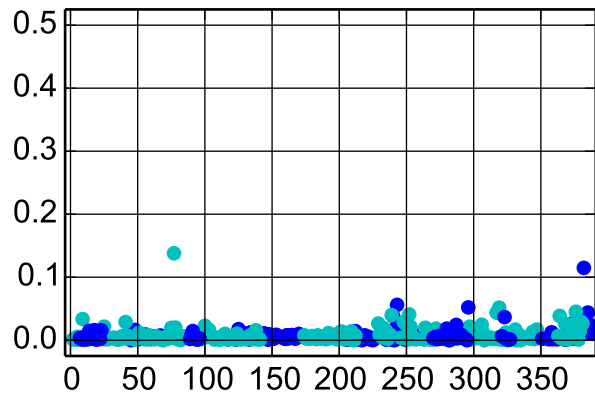

dbuz TIR 5.tsv : 5

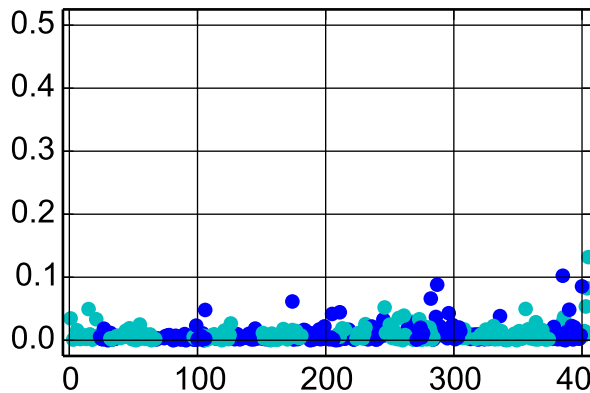

dbuz TIR 6.tsv : 6

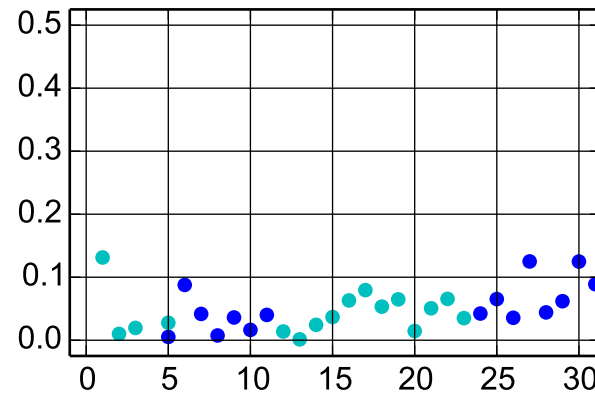

Supplement: Additional file 1 — Supplementary Figures. Supplementary Figure 1 (a to h). Chromosomal TE density. Main transposable element order density in 50 kb non-overlapping windows. Only mapped and oriented scaffolds are present, N90 scaffolds for D. buzzatii st-1 (a to d), and N80 scaffolds for D. mojavensis (e to h). Changes in dot colors denote scaffold changes. Supplementary Figure 2. D. buzzatii j-19 Order correction. Order contribution (kb) to D. buzzatii j-19 genome before (blue) and after (red) the coverage-based correction. Supplementary Figure 3. D. buzzatii j-19 Superfamily correction. Superfamily contribution (kb) to D. buzzatii j-19 genome before (blue) and after (red) the coverage-based correction. (ZIP 792 kb) [file 12864_2016_2648_MOESM1_ESM.zip › Supplementary_Figure_1c_Multiplot-dbuz_TIR.pdf]

dbuz LINE X.tsv : X

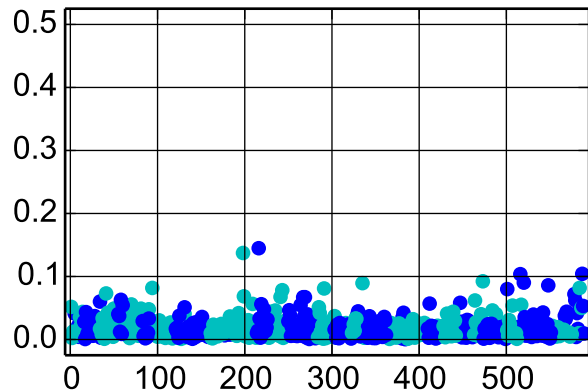

dbuz LINE 2.tsv : 2

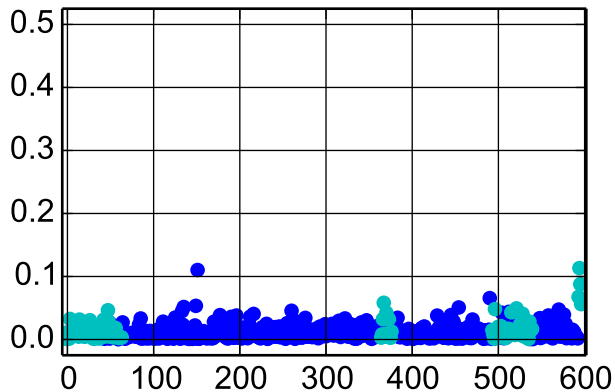

dbuz LINE 3.tsv : 3

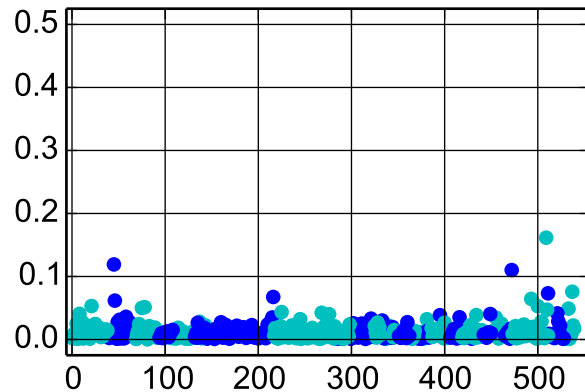

dbuz LINE 4.tsv : 4

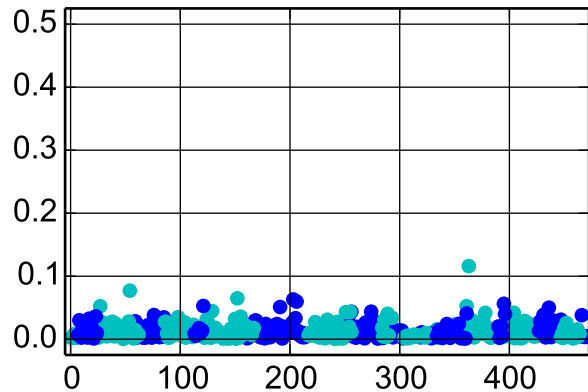

dbuz LINE 5.tsv : 5

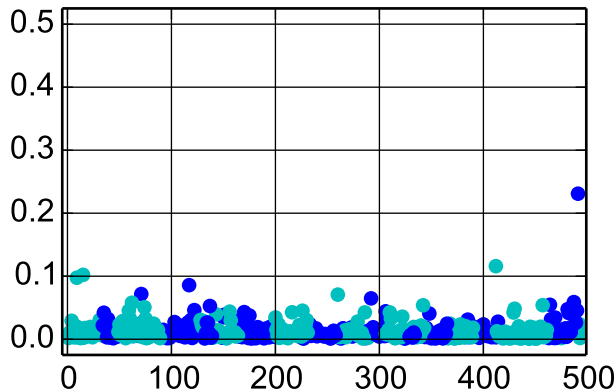

dbuz LINE 6.tsv : 6

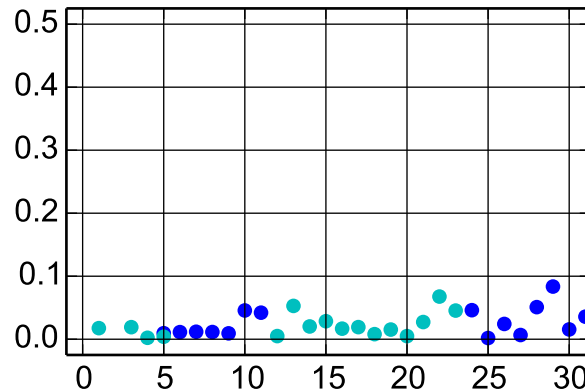

Supplement: Additional file 1 — Supplementary Figures. Supplementary Figure 1 (a to h). Chromosomal TE density. Main transposable element order density in 50 kb non-overlapping windows. Only mapped and oriented scaffolds are present, N90 scaffolds for D. buzzatii st-1 (a to d), and N80 scaffolds for D. mojavensis (e to h). Changes in dot colors denote scaffold changes. Supplementary Figure 2. D. buzzatii j-19 Order correction. Order contribution (kb) to D. buzzatii j-19 genome before (blue) and after (red) the coverage-based correction. Supplementary Figure 3. D. buzzatii j-19 Superfamily correction. Superfamily contribution (kb) to D. buzzatii j-19 genome before (blue) and after (red) the coverage-based correction. (ZIP 792 kb) [file 12864_2016_2648_MOESM1_ESM.zip › Supplementary_Figure_1b_Multiplot-dbuz_LINE.pdf]

dbuz LTR X.tsv : X

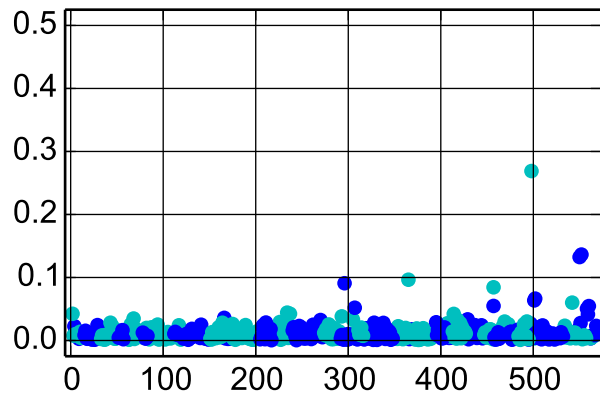

dbuz LTR 2.tsv : 2

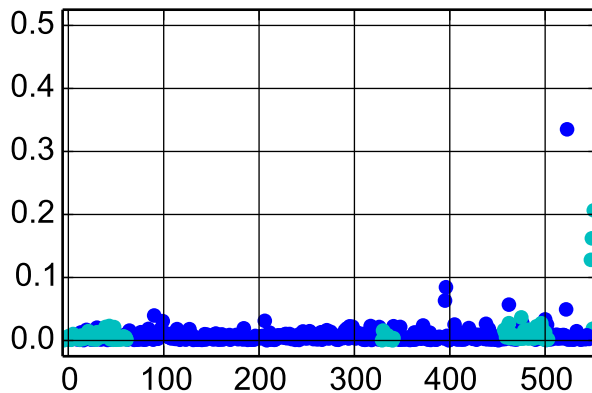

dbuz LTR 3.tsv : 3

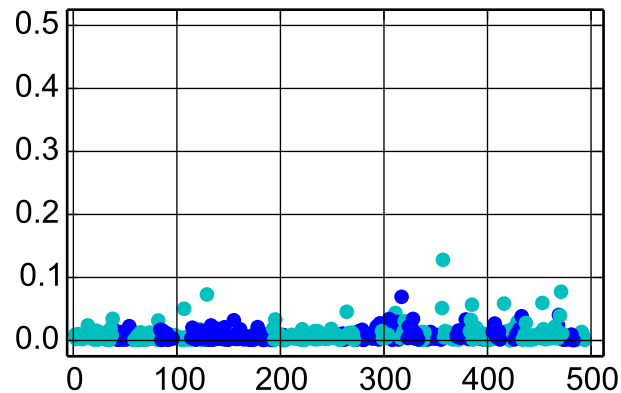

dbuz LTR 4.tsv : 4

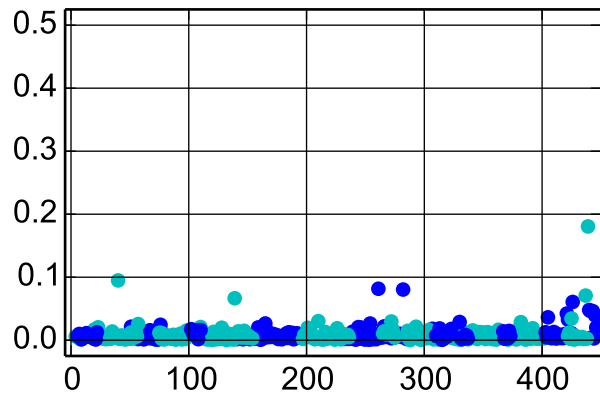

dbuz LTR 5.tsv : 5

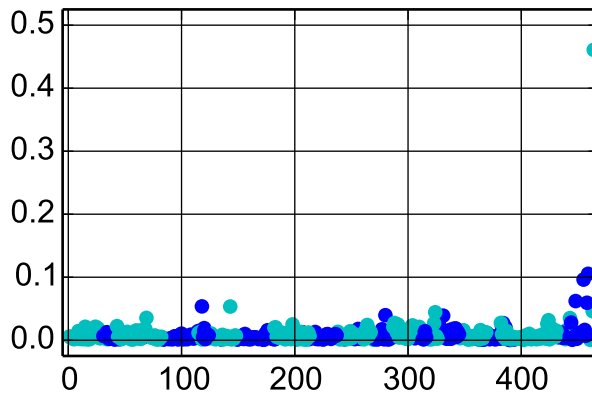

dbuz LTR 6.tsv : 6

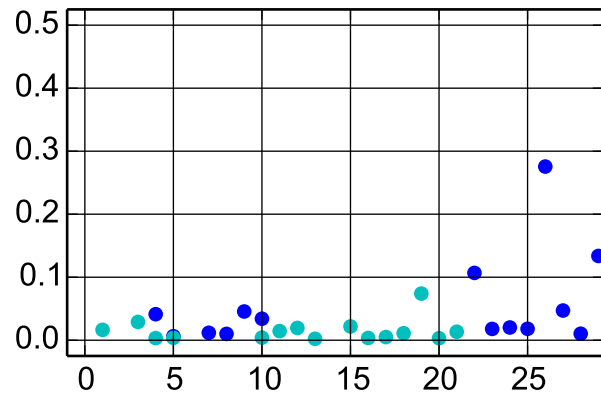

Supplement: Additional file 1 — Supplementary Figures. Supplementary Figure 1 (a to h). Chromosomal TE density. Main transposable element order density in 50 kb non-overlapping windows. Only mapped and oriented scaffolds are present, N90 scaffolds for D. buzzatii st-1 (a to d), and N80 scaffolds for D. mojavensis (e to h). Changes in dot colors denote scaffold changes. Supplementary Figure 2. D. buzzatii j-19 Order correction. Order contribution (kb) to D. buzzatii j-19 genome before (blue) and after (red) the coverage-based correction. Supplementary Figure 3. D. buzzatii j-19 Superfamily correction. Superfamily contribution (kb) to D. buzzatii j-19 genome before (blue) and after (red) the coverage-based correction. (ZIP 792 kb) [file 12864_2016_2648_MOESM1_ESM.zip › Supplementary_Figure_1a_Multiplot-dbuz_LTR.pdf]

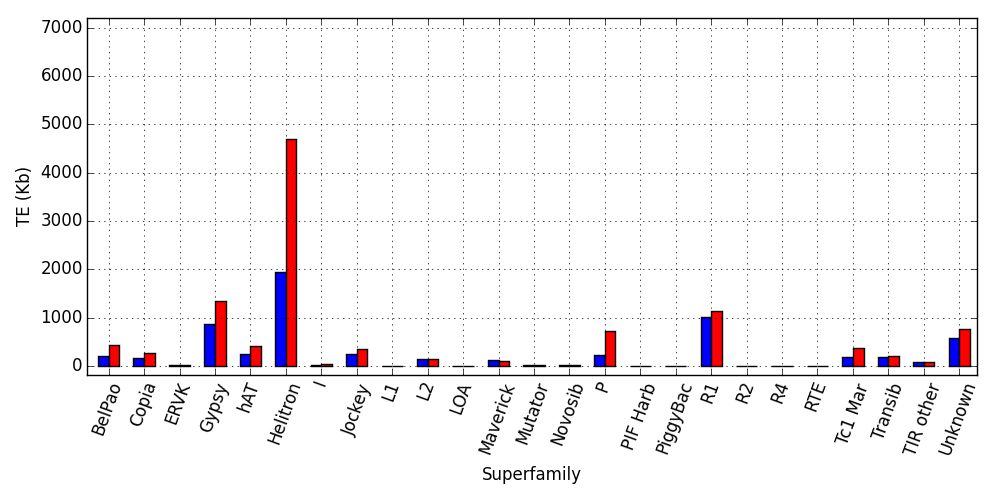

Supplement: Additional file 1 — Supplementary Figures. Supplementary Figure 1 (a to h). Chromosomal TE density. Main transposable element order density in 50 kb non-overlapping windows. Only mapped and oriented scaffolds are present, N90 scaffolds for D. buzzatii st-1 (a to d), and N80 scaffolds for D. mojavensis (e to h). Changes in dot colors denote scaffold changes. Supplementary Figure 2. D. buzzatii j-19 Order correction. Order contribution (kb) to D. buzzatii j-19 genome before (blue) and after (red) the coverage-based correction. Supplementary Figure 3. D. buzzatii j-19 Superfamily correction. Superfamily contribution (kb) to D. buzzatii j-19 genome before (blue) and after (red) the coverage-based correction. (ZIP 792 kb) [file 12864_2016_2648_MOESM1_ESM.zip › Supplementary_Figure_3.png]

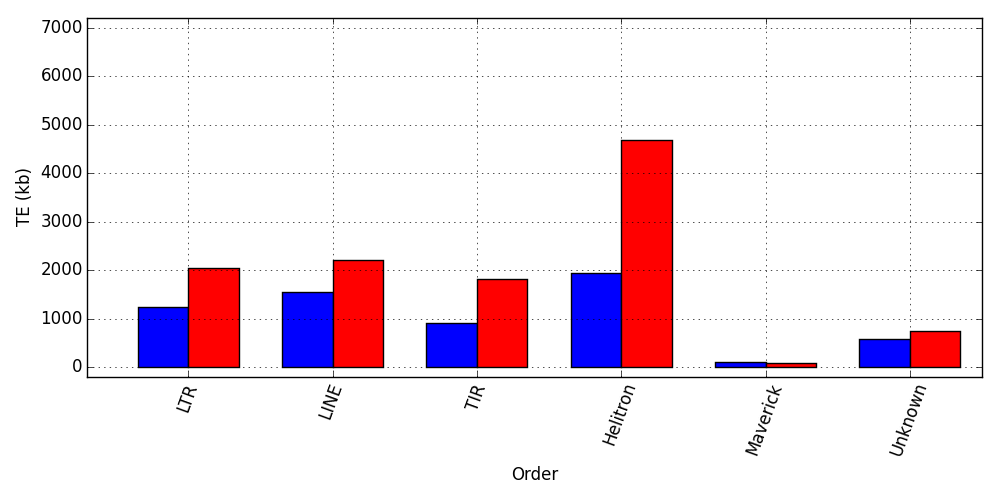

Supplement: Additional file 1 — Supplementary Figures. Supplementary Figure 1 (a to h). Chromosomal TE density. Main transposable element order density in 50 kb non-overlapping windows. Only mapped and oriented scaffolds are present, N90 scaffolds for D. buzzatii st-1 (a to d), and N80 scaffolds for D. mojavensis (e to h). Changes in dot colors denote scaffold changes. Supplementary Figure 2. D. buzzatii j-19 Order correction. Order contribution (kb) to D. buzzatii j-19 genome before (blue) and after (red) the coverage-based correction. Supplementary Figure 3. D. buzzatii j-19 Superfamily correction. Superfamily contribution (kb) to D. buzzatii j-19 genome before (blue) and after (red) the coverage-based correction. (ZIP 792 kb) [file 12864_2016_2648_MOESM1_ESM.zip › Supplementary_Figure_2.png]
