# Supplementary material for: Exploration of the Drosophila buzzatii transposable element content suggests underestimation of repeats in Drosophila genomes
Source: BMC Genomics. 2016 May 10;17:344. doi: 10.1186/s12864-016-2648-8 (PMC4862133; doi:10.1186/s12864-016-2648-8)
Supplement: Additional file 2 — Supplementary Tables. Supplementary Table 1. D statistics and p-values of U two-sample Kolmogorov-Smirnov tests comparing the distributions of TE densities in 50-Kb windows of each pair of chromosomes in three different sets: whole chromosome, central+distal and proximal regions. Only mapped and oriented scaffolds were considered. (PDF 389 kb) [file 12864_2016_2648_MOESM2_ESM.pdf]

Additional file 2. Supplementary tables 1 to 12

Table 1 D statistics of two samples Kolmogorov-Smirnov tests comparing the distributions of TE densities of the pairs *D. mojavensis* total

Total	*D. mojavensis*				
D-value	Chr2	Chr3	Chr4	Chr5	Chr6
ChrX	0.394	0.328	0.460	0.485	0.789
Chr2	-	0.087	0.083	0.103	0.837
Chr3	-	-	0.145	0.165	0.807
Chr4	-	-	-	0.051	0.822
Chr5	-	-	-	-	0.863

Table 2 p-values of two samples Kolmogorov-Smirnov tests comparing the distributions of TE densities of the pairs *D. mojavensis* total

Total	*D. mojavensis*				
p-value	Chr2	Chr3	Chr4	Chr5	Chr6
ChrX	0.000	0.000	0.000	0.000	0.000
Chr2	-	0.012	0.028	0.003	0.000
Chr3	-	-	0.000	0.000	0.000
Chr4	-	-	-	0.467	0.000
Chr5	-	-	-	-	0.000

Table 3 D statistics of two samples Kolmogorov-Smirnov tests comparing the distributions of TE densities of the pairs *D. buzzatii* total

Total	*D. buzzatiii*				
D-value	Chr2	Chr3	Chr4	Chr5	Chr6
ChrX	0.335	0.402	0.423	0.372	0.930
Chr2	-	0.085	0.117	0.063	0.947
Chr3	-	-	0.042	0.056	0.944
Chr4	-	-	-	0.064	0.946
Chr5	-	-	-	-	0.941

Table 4 p-values of two samples Kolmogorov-Smirnov tests comparing the distributions of TE densities of the pairs *D. buzzatii* total

Total	*D. buzzatiii*				
p-value	Chr2	Chr3	Chr4	Chr5	Chr6
ChrX	0.000	0.000	0.000	0.000	0.000
Chr2	-	0.019	0.001	0.187	0.000
Chr3	-	-	0.734	0.353	0.000
Chr4	-	-	-	0.247	0.000
Chr5	-	-	-	-	0.000

Table 5 D statistics of two samples Kolmogorov-Smirnov tests comparing the distributions of TE densities of the pairs *D. mojavensis* Central + Distal

Distal + Central	*D. mojavensis*			
D-value	Chr2	Chr3	Chr4	Chr5
ChrX	0.420	0.362	0.507	0.520
Chr2	-	0.088	0.111	0.117
Chr3	-	-	0.158	0.168
Chr4	-	-	-	0.047

Table 6 p-values of two samples Kolmogorov-Smirnov tests comparing the distributions of TE densities of the pairs *D. mojavensis* Distal + Central

Distal + Central	*D. mojavensis*			
p-value	Chr2	Chr3	Chr4	Chr5
ChrX	0.000	0.000	0.000	0.000
Chr2	-	0.018	0.002	0.001
Chr3	-	-	0.000	0.000
Chr4	-	-	-	0.645

Table 7 D statistics of two samples Kolmogorov-Smirnov tests comparing the distributions of TE densities of the pairs *D. buzzatii* Distal + Central

Distal + Central	*D. buzzatiii*			
D-value	Chr2	Chr3	Chr4	Chr5
ChrX	0.354	0.431	0.474	0.404
Chr2	-	0.095	0.144	0.080
Chr3	-	-	0.059	0.053
Chr4	-	-	-	0.080

Table 8 p-values of two samples Kolmogorov-Smirnov tests comparing the distributions of TE densities of the pairs *D. buzzatii* Distal + Central

Distal + Central	*D. buzzatiii*			
p-value	Chr2	Chr3	Chr4	Chr5
ChrX	0.000	0.000	0.000	0.000
Chr2	-	0.011	0.000	0.063
Chr3	-	-	0.365	0.494
Chr4	-	-	-	0.106

Table 9 D statistics of two samples Kolmogorov-Smirnov tests comparing the distributions of TE densities of the pairs *D. mojavensis* Proximal

Centromeric	*D. mojavensis*			
D-value	Chr2	Chr3	Chr4	Chr5
ChrX	0.297	0.412	0.166	0.480
Chr2	-	0.417	0.183	0.317
Chr3	-	-	0.400	0.683
Chr4	-	-	-	0.383

Table 10 p-values of two samples Kolmogorov-Smirnov tests comparing the distributions of TE densities of the pairs *D. mojavensis* Proximal

Centromeric	*D. mojavensis*			
p-value	Chr2	Chr3	Chr4	Chr5
ChrX	0.008	0.000	0.356	0.000
Chr2	-	0.000	0.239	0.004
Chr3	-	-	0.000	0.000
Chr4	-	-	-	0.000

Table 11 D statistics of two samples Kolmogorov-Smirnov tests comparing the distributions of TE densities of the pairs *D. buzzatii* Proximal

Centromeric	*D. buzzatiii*			
D-value	Chr2	Chr3	Chr4	Chr5
ChrX	0.138	0.171	0.158	0.151
Chr2	-	0.179	0.128	0.227
Chr3	-	-	0.121	0.207
Chr4	-	-	-	0.224

Table 12 p-values of two samples Kolmogorov-Smirnov tests comparing the distributions of TE densities of the pairs *D. buzzatii* Proximal

Centromeric	*D. buzzatiii*			
p-value	Chr2	Chr3	Chr4	Chr5
ChrX	0.609	0.337	0.437	0.498
Chr2	-	0.280	0.694	0.083
Chr3	-	-	0.765	0.146
Chr4	-	-	-	0.093
